# Automated pipeline for leaf spot severity scoring in peanuts using segmentation neural networks

**DOI:** 10.1186/s13007-024-01316-x

**Published:** 2025-02-20

**Authors:** Joshua Larsen, Jeffrey Dunne, Robert Austin, Cassondra Newman, Michael Kudenov

**Affiliations:** 1 Department of Electrical and Computer Engineering, NC State University, 890 Oval Dr, Raleigh, NC 27606 USA; 2 Department of Crop and Soil Science, NC State University, 101 Derieux Pl, Raleigh, NC 27695 USA; 3 Plant Science Initiative, NC State University, 840 Oval Dr, Raleigh, NC 27606 USA

**Keywords:** Automation, Phenotyping, Peanut, Leaf spot, Imaging, Computer vision, Neural network

## Abstract

**Background:**

Late and early leaf spot in peanuts is a foliar disease contributing to a significant amount of lost yield globally. Peanut breeding programs frequently focus on developing disease-resistant peanut genotypes. However, existing phenotyping protocols employ subjective rating scales, performed by human raters, who determine the severity of leaf spot infection. The objective of this study was to develop an objective end-to-end pipeline that can serve to replace an expert human scorer in the field. This was accomplished using image capture protocols and segmentation neural networks that extracted lesion areas from plot-level images to determine an appropriate rating for infection severity.

**Results:**

The pipeline incorporated a neural network that accurately determined the infected leaf surface area and identified dead leaves from plot-level cellphone imagery. Image processing algorithms then convert these labels into quality metrics that can efficiently score these images based on infected versus non-infected area. The pipeline was evaluated using field data from plots with varying leaf spot severity, creating a dataset of thousands of images that spanned conventional visual severity scores ranging from 1–9. These predictions were based on the amount of infected leaf area and the presence of defoliated leaves in the surrounding area. We were able to demonstrate automated scoring, as compared to expert visual scoring, with a root mean square error of 0.996 visual scores, on individual images (one image per plot), and 0.800 visual scores when three images were captured of each plot.

**Conclusion:**

Results indicated that the model and image processing pipeline can serve as an alternative to human scoring. Eliminating human subjectivity for the scoring protocols will allow non-experts to collect scores and may enable drone-based data collection. This could reduce the time needed to obtain new lines or identify new genes responsible for leaf spot resistance in peanut.

## Background

Peanut is an economically important agricultural crop that is grown and consumed world-wide. It is considered a sustainable source of protein due to its ability to grow in soils that are difficult for other crops while also requiring less water than tree nuts [1]. With over 1.6 million acres of peanuts being grown annually in the United States alone, it is a major contributor to the food supply in the United States [2]. Early and Late Leaf Spot, caused by Passalora arachidicola and Nothopassalora personata, are often considered the most critical yield-reducing diseases in peanut [3]. Yield loss caused by leaf spot can be over fifty percent [4]. Estimates place the lost yield as high as $600 million annually, where fungicide control costs can be as high as $250 per hectare [5]. The breeding of peanut varieties that are naturally resistant to this highly destructive disease is key to many peanut breeding programs worldwide [6–9].

The most common method to determine the severity of infection is through a field survey [10]. Here, individual plots within a field trial will be evaluated or “scored” by one of the expert raters who perform the field trials. Scoring methods typically involve a set of subjective guidelines that an expert follows to rate the severity of infection, such as the Florida 1–10 scale as outlined by Chiteka et al. or the modified 9-point scale as described by Subrahmanyam et al. [6, 11]. These scales have been used extensively by research teams in breeding programs due to their ease of use [12–15]. These scales are nondestructive as they are based solely on visual symptoms; however, it contains human biases. This human element in the scoring process makes the results difficult to replicate and can lead to different analyses of the same genotypes and severities. Collaboration between breeders is made difficult due to this source of error, such that correcting the cause of discrepancies between visual severity scores becomes largely impractical. It can be difficult to identify what variations in severity score between two trials of the same genotype are caused by human subjectivity in scoring metrics instead of true variations in the response to leaf spot by the different trials.

Due to difficulty in scoring, new methods of disease detection have evolved within the realm of remote sensing. For example, the use of canopy hyperspectral reflectance measurements has proven to be a reliable method for the detection of leaf spot within peanut as well as many other types of crop pathogens [10, 16–20]. These methods require specialized sensing equipment, specifically hyperspectral or infrared imaging systems, which can be expensive and difficult to use properly. Beyond hyperspectral imaging systems, computer vision approaches have become popular for the purpose of classification. These developments offer new capabilities for detection that is more similar to human vision approaches. Outside of the agricultural world, these types of deep learning networks have been used extensively for damage detection in subjects ranging from large buildings to small semiconductor wafers [21–24]. New styles of convolutional neural networks have achieved high accuracy when used for the classification of plants and their associated diseases [25]. Numerous existing networks can be used for transfer learning, facilitating the process of developing a neural network for classification [26]. Well-made convolutional networks for this type of task can identify the texture and color of the disease, which ensures that the network is properly identifying disease symptoms present in the image [27]. Given that one of the key metrics used in scoring leaf spot severity is the percentage infected region, a neural network capable of identifying leaf spot lesions could be used to determine infection severity, as has been attempted in phenotyping pipelines previously [6, 28].

Disease presenting as a form of visual sore on vegetation is not unique to Peanut, with recent research focusing on development of segmentation networks for disease detection in agriculture. Recent work has shown that lightweight neural networks are capable of being trained to properly segment disease across a range of agricultural settings [29]. For instance, other recent work has found that disease segmentation can be used to detect Tar Spot disease in corn with the goal of tying the outputs of the segmentation network to a traditional disease scale [ahamd2024tar]. A similar convolutional network based pipeline for disease severity determination in plum has also been developed [30]. Peanut and other groundnut have also been the subject of disease detection and severity determination pipelines. Chapu et. al. developed a pipeline capable of classifying leaf spot disease severity using a number of camera systems and models that classify severity based on color indices and normalized vegetation indices [31]. Similarly, Lin et al. developed a system for detecting individual leaf spot instances, counting the number of lesions on in-field peanut using an automated image-capturing system capable of navigating the field [32]. However, neither prior pipeline has (1) validated against the conventional 9-point scale scale and visual scores; and (2) has incorporated instance segmentation to provide quantitative ratios of diseased to healthy leaf areas; and (3) has been validated over multiple years of data at multiple trial locations. By incorporating the segmentation networks seen with other disease monitoring systems and expanding the rigor of the dataset, it is possible to develop a leaf spot severity scoring system that more closely resembles the process of a traditional breeding pipeline. The outputs of the neural network allows for the pipeline to be used with the existing 9-point scale and also independently, something prior neural networks and disease scoring pipelines did not incorporate.

To resolve the aforementioned issues related to subjectivity in leaf spot grading and lower the barrier of entry to automated leaf spot scoring, the newly developed pipeline uses handheld cameras to image peanuts which have been exposed to leaf spot pathogens. These images are processed by a semantic segmentation neural network where the ratios of healthy, infected, and dead plant material is extracted as an intermediary step. A simple fitting function is then used to convert these values to a ratio of plant material that has been infected or destroy by leaf spot. Finally, this value is converted to the more traditional 9-point scale [11]. This process serves to minimize the human subjectivity error present during scoring of leaf spot severity while building from the 9-point scale to preserve the traditional interpretation of leaf spot severity scoring. Compared to other automated leaf spot scoring pipelines, which only return an estimated 9-point scale score or information related to the number and size of lesions, this pipeline generates both the intermediary ratios of infected, dead, and healthy leaf area as well as a score in accordance with the 9-point scale, which ensures that this pipeline can meet the needs of researchers and breeders regardless of how they choose to score severity [31, 32].

This paper is structured as follows: "Methods" section describes the materials and methodology employed during this study. It details the choice of camera equipment used, the software used in developing this pipeline, and how the performance was validated. "Results" section details the results of this pipeline, showcasing the accuracy to which it can predict the disease severity based when the protocols established in "Methods" are followed. "Discussion" section provides a discussion of the limitations of this study and a discussion of how well the results resolve the problems originally described, and a as proposal of future work to improve upon the results seen here. Finally, "Conclusion" section is a brief conclusion, reestablishing the goals of this project and describing how well the original goals were accomplished.

## Methods

**Fig. 1:**
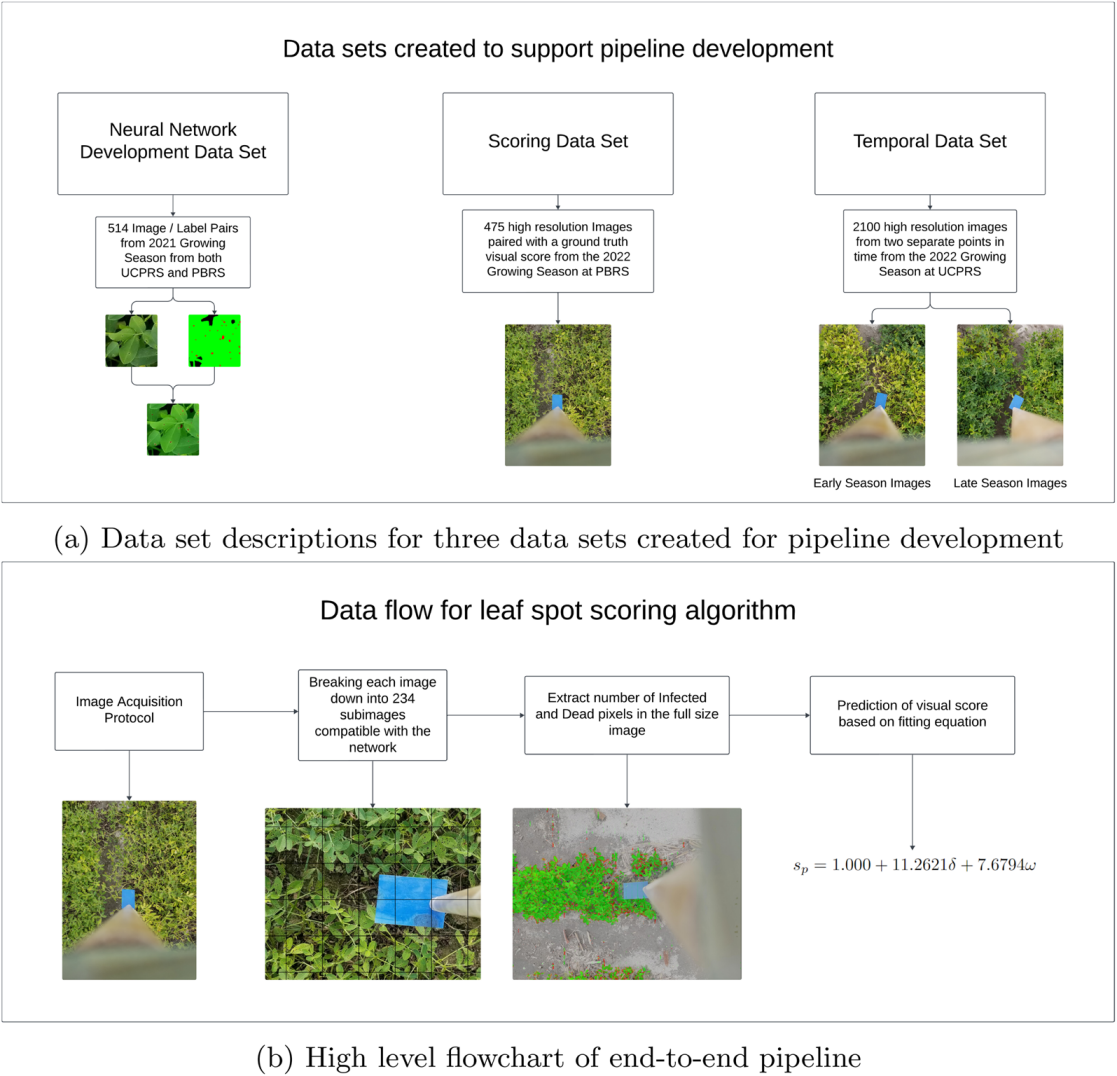
Overview of (**a**) data sets created to support this pipeline’s development, and (**b**) the overall data flow when executing the pipeline

The process for developing the end-to-end pipeline consisted of two phases, (1) using data collected during the 2021 growing season to develop the trained segmentation network and (2) employing this new network on data collected during the 2022 growing season to finalize the conversion from network output to scored severity. The datasets from 2021 and 2022 were kept separate to ensure the generalizability of the model and to prevent over fitting to specific year-related patterns. Figure 1 shows the general data flow that was used during each phase of the project.

### Field trials

Data was collected over two years, across two locations. The first location was the Peanut Belt Research Station (PBRS) near Lewiston, North Carolina, located at 36.1333, − 77.1705. The second location was the Upper Coastal Plain Research Station (UCPRS) near Rocky Mount, North Carolina, situated at 35.8943, − 77.6812. A total of 2120 plots were grown for the purpose of studying leaf spot over the course of the 2021 and 2022 growing seasons. Field trials consisted of 530 plots, with each trial containing 265 different genotypes of peanut to be monitored. Each plot consisted of two rows of a single line. Rows were approximately 0.9 meters in width and 7.6 meters in length. Further information regarding planting, visual scoring, and image capture dates can be found in Table 1 and a complete description of the trials can be found in [20].

### Image acquisition protocol

**Fig. 2:**
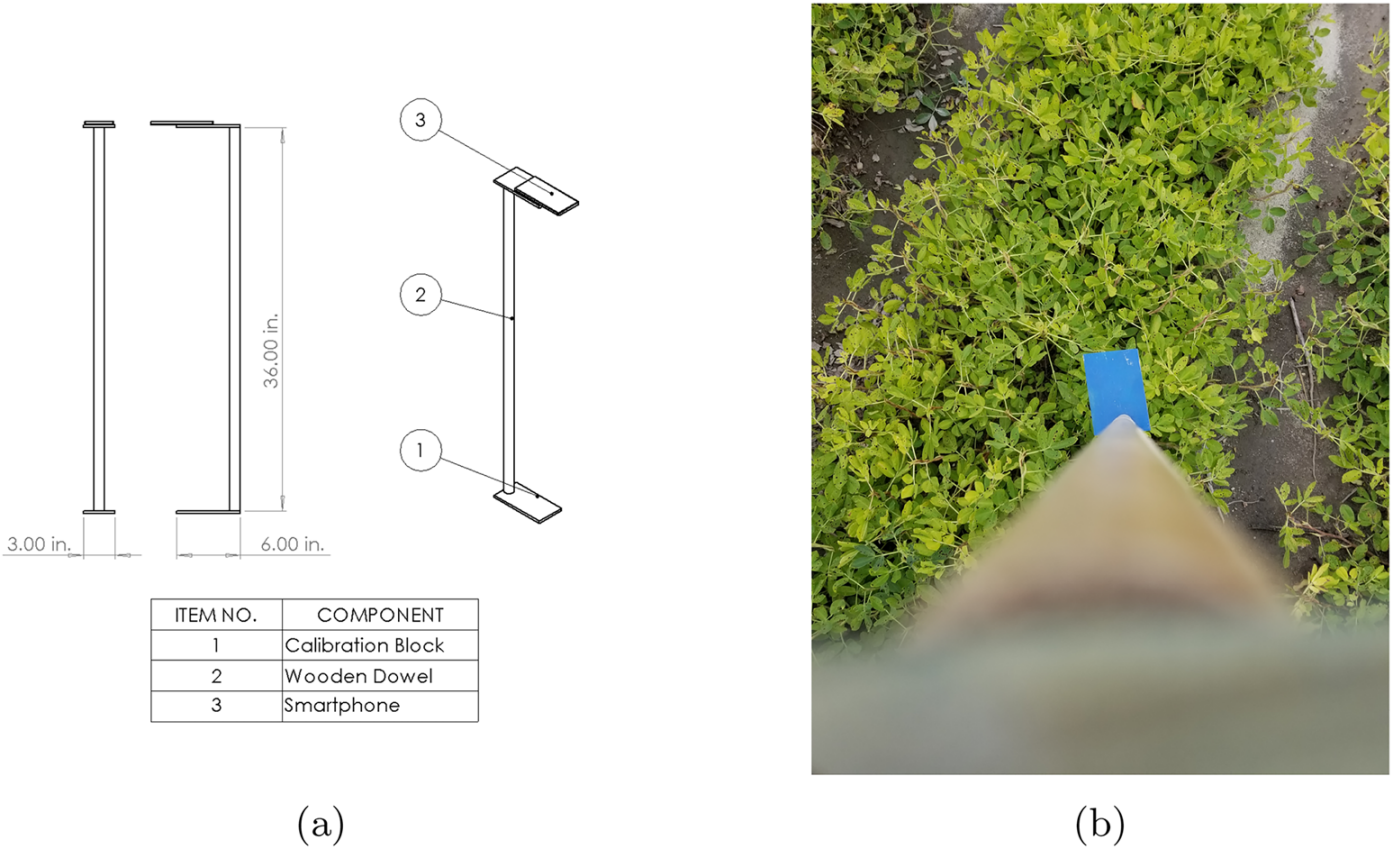
Cell phone **a** image acquisition configuration; and **b** example of acquired image of the canopy and calibration target

To facilitate image acquisition and development of necessary data sets, a protocol was developed to image in the field. To ensure that the canopy was at a consistent distance (3 ft) from the camera, handheld mounts were constructed to serve as references. A schematic of this simple setup is shown in Fig. 2a in which a 36-inch wooden dowel was combined with a small phone mount. A spatial calibration block was fixed to the end of the dowel; This calibration block was captured in all images and ensured that a spatial reference was included in all images, allowing the determination of the real world size of objects in the images [33–35]. Per Fig. 2b, this configuration allowed the user to hold the mount so that the reference block rested just above the canopy while the phone was positioned at the opposite end of the dowel. The pixel sampling distances in each image were converted to physical distance by $d=a/b$, where *d* is the calibrated sampling distance, *a* is the width of the block in pixels, and *b* is the width of the block in pixels. For this setup, the block had a width of 1 inch, and generally the camera was oriented such that $b=282$ pixels, so these images had an approximate sampling distance of 282 pixels / inch. These images were 3042 x 4032 pixels in size, the maximum resolution of the Samsung Galaxy S8 phones used. The effective focal length of this camera was 24 mm. Employing a wide angle lens ensures that off-axis regions are included in the captured images, ensuring that more than just the highest leaves are imaged. All images were captured on days with minimal cloud cover in the early afternoon, ensuring that consistent lighting was present in all images.

A minimum of three images per plot were captured. The first and last images were captured at the extreme ends of the length of the plot, with the image arranged to capture the edge of the plot while minimizing non-plant area in frame. Often, more than the minimum number was taken as the imaging personnel walked from one end of the plot to the other. This ensured good coverage was maintained while also allowing each plot to be rapidly imaged. An example of these images can be seen in Fig. 2b. Table 1 contains the dates at which the imaging protocol was performed. On dates image capture took place, a random number generator was used to select a subset of plots for imaging.

**Table 1:** Collection of planting, harvesting, and imaging dates

2021 Growing season			2022 Growing season		
	UCPRS	PBRS		UCPRS	PBRS
Planting dates	3-May-21	15-May-21	Planting dates	9-May-22	28-Apr-22
Harvesting dates		22-Oct-21	Harvesting dates		
Imaging dates	15-Jul-21	30-Sep-21	Imaging dates	29-Sept-22	5-Oct-22
	8-Oct-21			11-Oct-22	
	11-Oct-21				

**Fig. 3:**
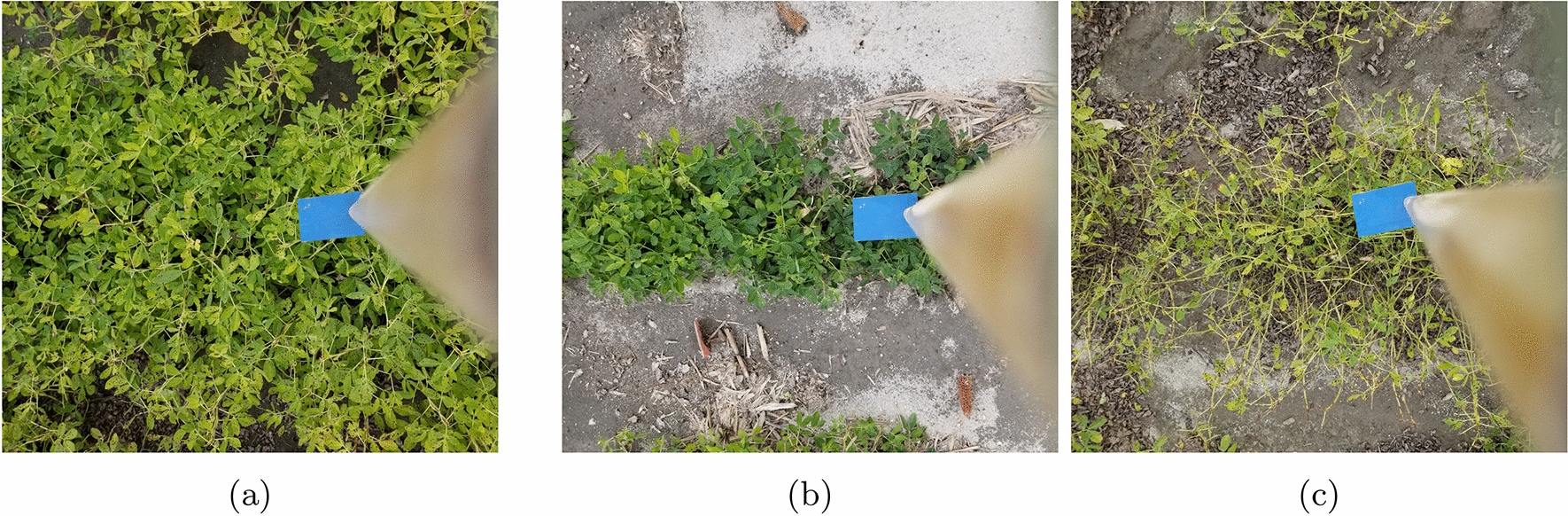
Visualization of peanut of varying leaf densities and plot thickness. Each of these images was captured during field trials and used as part of the *Image Scoring Data Set* detailed in Transfer learning and selecting the neural network architecture section

A goal of having included many different peanut lines at different times in the growing season was to ensure that the data set included peanuts with different canopy structures, specifically as it relates to leaf density. An example of images that showcase different structures can be found in Fig. 3. These images were used in the development process of this pipeline as part of the Image Scoring Data Set described in Transfer learning and selecting the neural network architecture section.

### Data sets created for pipeline development

Three primary data sets were created over the course of this study, each with a different purpose. The first will be referred to as the *Neural Network Training Data Set*. This data set was made of images captured at both UCPRS and PBRS locations during the 2021 growing season. All of the 2021 imaging dates found in Table 1 were included in this data set. The final result of this data set was 514 image / semantic segmentation labels that were used for developing the neural network used in the pipeline. The second data set is the *Image Scoring Data Set*. This data consists of 476 images that were collected at the PBRS location on 5 Oct. 2022 and includes data from plots that were manually scored from an infection level 1 to an infection level 9. This data set was used to develop the scoring function and model its success against a manual ground truth scoring by a trained expert. The last major data set is the *Temporal Data Set*. This data set includes randomly sampled field trials toward the end of the 2022 growing season. Two, subsets of this data set were created by randomly imaging plots from the UCPRS location in accordance with the imaging protocol. The first subset was collected at the UCRPS location on 29 September 2022 and contained 1,434 images. The second data set was collected at the same UCPRS location on 11 October 2022 and contained 714 images. Both subsets involved a different set of randomly sampled plots with 118 plots in total imaged for the *Temporal Data Set*.

#### Semantic segmentation labeling

Image processing started by randomly cropping the raw images to a smaller dimension of $224\times 224$ pixels. These dimensions are commonly seen in many neural network architectures, such as ResNet50 and MobileNet [36, 37]. All training images were cropped randomly from the fill size raw images using a simple MatLab script that selected a single random crop from each of the images from the 2021 growing season. The crops were taken randomly by using a random number generator to choose an upper left pixel for the subimage. The three remaining corners were calculated and the subimage was then cropped out of the larger image. These cropped subimages were stored in a local directory as individual images to facilitate the training process.

**Fig. 4:**
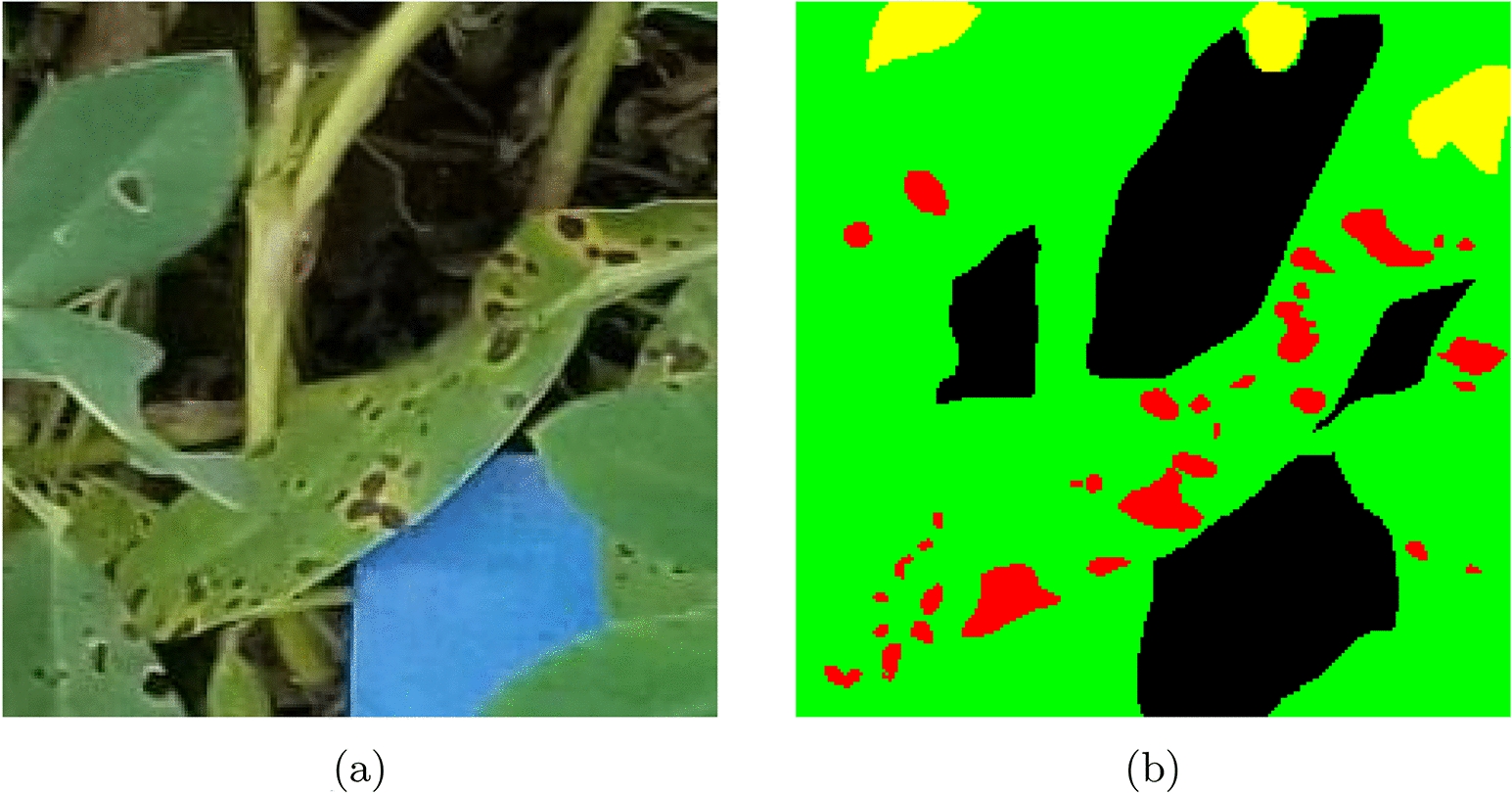
Comparison of **a** an image patch versus **b** its semantic segmentation label, where red represents infected lesion area, green represents the healthy subclass, yellow represents dead or defoliated plant material, and black represents non-plant / non-discernible area

Using the Labelbox platform, labels for semantic segmentation were generated on hundreds of these patches [38]. At the pixel level, the images were divided into one of four classes: (1) healthy, or uninfected green leaf area that shows no signs of leaf spot infection; (2) infected, which represents living but discoloured leaf tissue caused by the leaf spot infection; (3) dead, which is comprised of non-living and defoliated plant tissue; and (4) non-plant, which describes any pixel capturing non-plant or indiscernible material. Due to the three dimensional canopy structure of peanut, it is possible that some area becomes obscured due to shadows cast from the upper canopy. This behavior was observed during the labeling process by our team; to resolve this, any regions that were obscured due to shadows cast part of the canopy structure was considered “non-plant” material for the purpose of semantic segmentation. Upon completing each image label, Labelbox saved a semantic label image that can be paired with its associated RGB image during training. Figure 4 shows one of these hand-made semantic label images. The complete data set containing the RGB images and the labels is available on a GitHub repository^1^.

### Transfer learning and selecting the neural network architecture

A neural network was transfer learned to perform semantic segmentation, such that each pixel would be classified as either healthy, infected, dead, or non-plant material. Four networks were chosen and trained prior to down-selecting the best-performing model. The U-Net architecture was first chosen given its origin in biomedical image segmentation and powerful encoder-decoder structure [39]. Three additional deep learning networks were also employed: MobileNet, ResNet18, and ResNet50 [36, 37]. These were implemented using DeepLabV3+ in MatLab, allowing the networks to all perform pixel-wise classification on the images [40].

The training data subset consisted of 420 image and semantic segmentation label pairs. The validation and holdout data subsets were 48 images each, representing a split of 81.4% / 9.3% / 9.3% for the training / validation / holdout split from the original *Neural Network Development data set*. Table 2 describes the raw pixel counts of each of the individual classes in the training data set as well as the normalized frequency for the classes.

**Table 2:** Raw counts and normalized values

	Raw counts	Normalized frequency
Healthy	15,439,914	0.7341
Infected	440,205	0.0209
Non-plant	3,848,687	0.183
Dead	1,303,985	0.062

All four networks were trained using the following settings:

1. Use of the Adam optimizer and a mini-batch size of 32 [41]. Note that mini-batch sizes of 8, 16, 32, and 128 were studied. A batch size of 32 resulted in the highest mean accuracy when training the network for 20 epochs with training parameters identical to the full model - the only change was the early stopping parameter was removed. Table 5 contains a brief summary of all mini-batch sizes that were studied and their finalized result metrics when evaluating the same validation subset as the finished model. 32 was the selected value for the mini batch size due to the maximum mean accuracy found with this testing.
2. Training parameters, summarized in Table 4, were identical for all four networks.
3. The training process spanned a dynamically chosen number of epochs based on an early stopping procedure. Note that while a maximum of 1500 epochs was technically possible and validation patience was 150 epochs, the actual number of training epochs was much lower [42–46]. MobileNetV2, ResNet18, ResNet50, and U-Net reached their minimum validation error at 4, 5, 12, and 60 epochs respectively, and these were the final models that were evaluated. Each model was trained for a further 150 epochs (although this overtrained version was not saved) to ensure that the model with minimum validation error had been selected.
4. The risk of over fitting was minimized by dividing the data into 81.4% training, 9.3% validation, and 9.3% holdout. All training, validation, and holdout data sets were comprised exclusively of crops taken from images from the 2021 growing season, although both field trials were used to create the model.
5. Class weights were incorporated into the loss function. The original data set suffered from a high degree of imbalance as shown in Table 2. Class weights, summarized in Table 3, were used to correct for this in each network, as weighting the classes should result in a more reliable performance on the unbalanced data set [47].
6. GPU acceleration was used to train the models at a faster rate than traditional CPU training. System specifications included an RTX 3060 12GB, a Ryzen 5 5600X, and 32GB of system RAM.

**Table 3:** Class weights for training

Class weights			
Healthy	Infected	Background 3	Dead
0.1966	1.1133	1.0417	0.9651

**Table 4:** Model training settings

Setting	Value
Initial learning rate	0.001
Learn rate schedule settings	None
L2 Regularization	0.0001
Gradient decay factor	0.9
Squared gradient decay factor	0.999
Epsilon	1e−8
Batch normalization statistics	Population

**Fig. 5:**
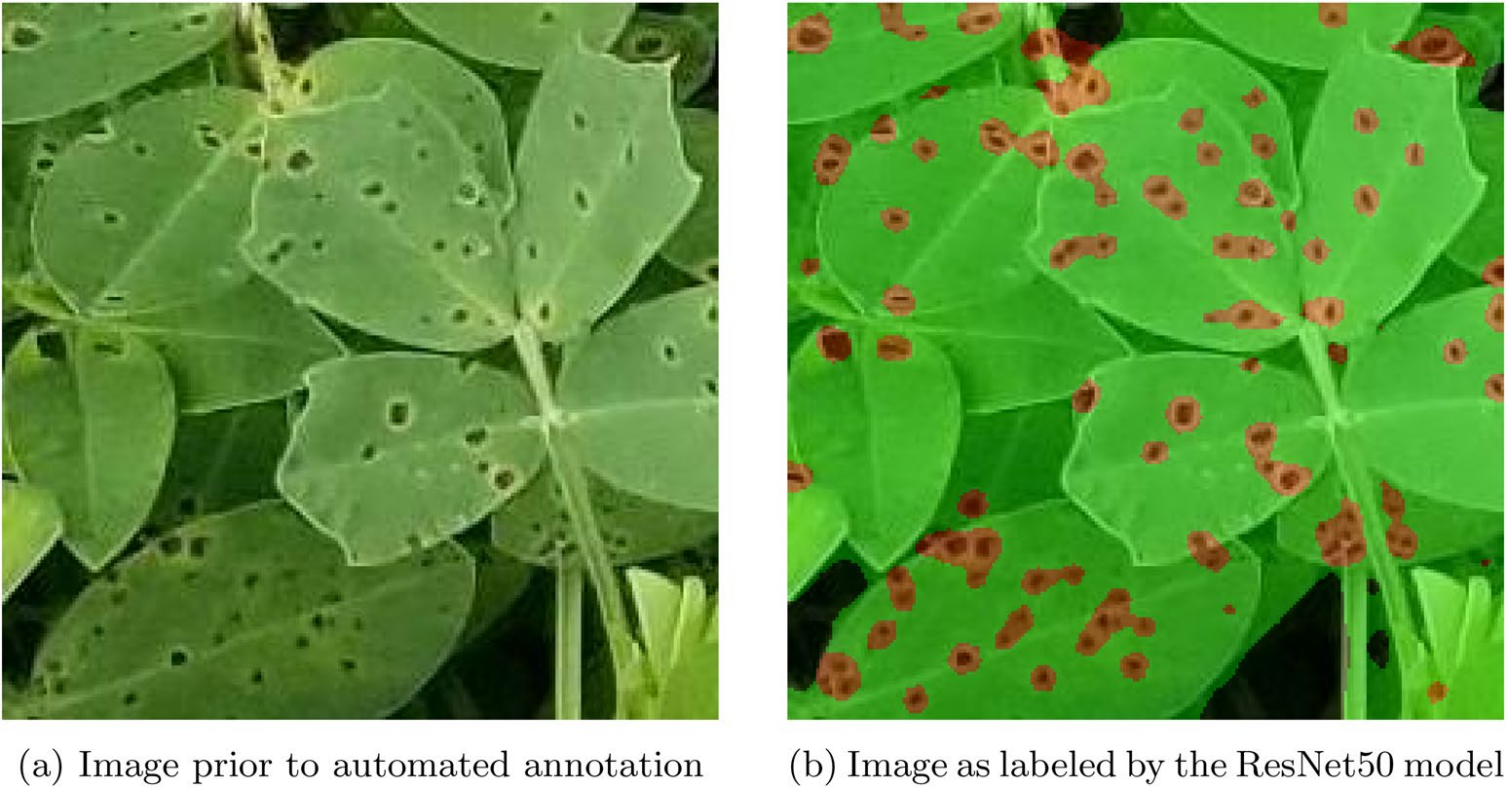
Comparison of an image patch versus its generated label. Green areas represent Healthy regions, red represents infected lesions or Infected, and black regions represent background or Non-Plant material. No completely dead material can be seen in this example

**Table 5:** Evaluation criteria on the validation subset with a ResNet Model Trained with varying mini batch size

Mini batch size	Global accuracy	Mean accuracy	Mean IoU	Weighted IoU
8	0.8038	0.68738	0.42089	0.7239
16	0.85449	0.85598	0.56597	0.78873
32	0.82879	0.872332	0.54744	0.75412
128	0.76852	0.76271	0.45586	0.68135

## Results

### Imaging protocol

The main metric for success with respect to the imaging protocol was the number of images captured and plots imaged compared to the speed of a human scorer. To that end, imaging per plot takes an individual approximately 1 minute. For a field of 530 plots, this would take almost 9 hours for an individual to properly image for scoring protocols. By comparison, an individual scoring in compliance with the traditional 9-point can score the same number of field trials in approximately 1–1.5 hours. To this end, the imaging protocol was much less time efficient than traditional field surveys, with imaging taking almost five times longer compared to a traditional field survey.

We also see a reduction in total plant canopy area used for scoring through the imaging protocol as described. Given the typical ground sampling distance of 94 pixels / inch, we are left with an approximate 0.90 $m^2$ area per image taken. Compared to a human scorer who can see the entirety of the 6.8 $m^2$ plot at a glance, this is a massive reduction in sampled area.

### Neural network performance

As previously described, a holdout data set of 48 image / segmentation label pairs was used to evaluate how well the models generalized to new information. The performance on this data set would demonstrate how the model performs on future new data sets. The hold out data set was used to generate two primary metrics of evaluation. The first metric was the Jaccard Index, which evaluates the similarities in the semantic segmentation labels versus the generated labels by comparing the intersection areas of each class to the overlapping areas of each class. The ResNet50 model scored the highest, with a Jaccard index of 0.770. A full list of Jaccard indices for each network, alongside global accuracy, can be seen in Table 6. The time for each model to perform inference was determined by running each model on one thousand $224\times 224$ pixel images, with the mean time to perform interference recorded. The GPU utilization was logged during this using the program GPU-Z version 0.8.9, with the mean GPU Utilization during this time period recorded for each model. Each of these tasks were performed using the aforementioned system with Ryzen 5600X CPU and RTX 3060 graphics card.

**Table 6:** Accuracy, Jaccard Index, inference time, and size of each model

Model	Mean accuracy	Jaccard Index	Inference time (ms)	Size (MB)	GPU utilization (%)
MobileNet	0.853	0.716	7.64	27.9	39.47
U-Net	0.815	0.721	17.7	124	81.96
ResNet18	0.795	0.665	5.28	82.8	51.18
ResNet50	0.838	0.770	7.92	177	56.21

**Fig. 6:**
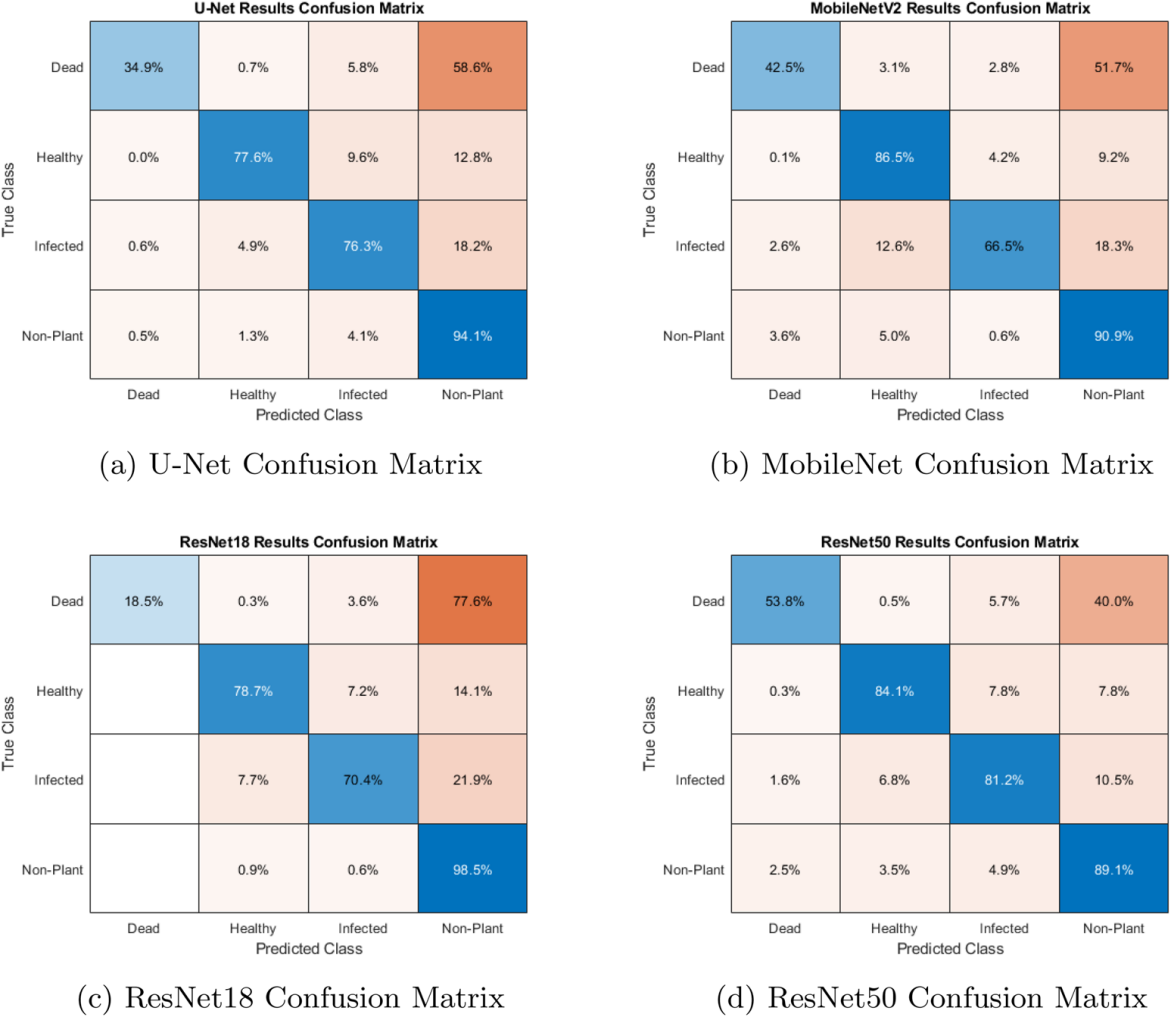
Confusion matrices for the four networks

The normalized Confusion Matrices for all four of the preliminary networks can be seen in Fig. 6, which shows how well the architectures could identify each pixel belonging to each class. While each model demonstrated a mean accuracy of between 0.795 and 0.853, the minimum performance of each model across the four classes was the metric used to decide which model would be used in the finished pipeline. Despite a larger model and longer inference time compared to the three other models that were tested, ResNet50 was the only network to achieve an accuracy of over 50% on the dead leaf material during testing (See Figure 5d). ResNet50 was selected for the application due to its highest minimum accuracy across classes. While mean accuracy and even Jaccard index indicates similar performance of each network, we determined that ResNet50 provides a good balance of all classes, spanning early leaf spot (visual scores 1–5) and defoliation (visual scores 6–9) based on the confusion matrices.

In addition to simple numeric testing of the network’s accuracy on test sets, it was important to perform visual inspections of how well the model generalized to new data. An example of this qualitative testing can be seen in Fig. 5, which shows an image that was passed into the preliminary ResNet50 architecture and its resulting label. As depicted, semantic labels were applied correctly, demonstrating proper semantic segmentation of the image—this is supported by the confusion matrix. Within the image, green represents Healthy plant area, red sections are infected lesions, yellow representing Dead plants, and black represents Non-Plant or non-distinguishable area.

All of the associated networks exhibited the lowest correct classification on the “Dead” class. ResNet50 saw the best result here, with more than half of the Dead pixels being properly classified. The other networks all saw under 50% proper classification of the Dead classed pixels, reinforcing our choice to keep the ResNet50 network in the pipeline. This low classification rate was noticeably exacerbated in images where there was no green plant area - this behavior was seen routinely.

### Scoring function

**Fig. 7:**
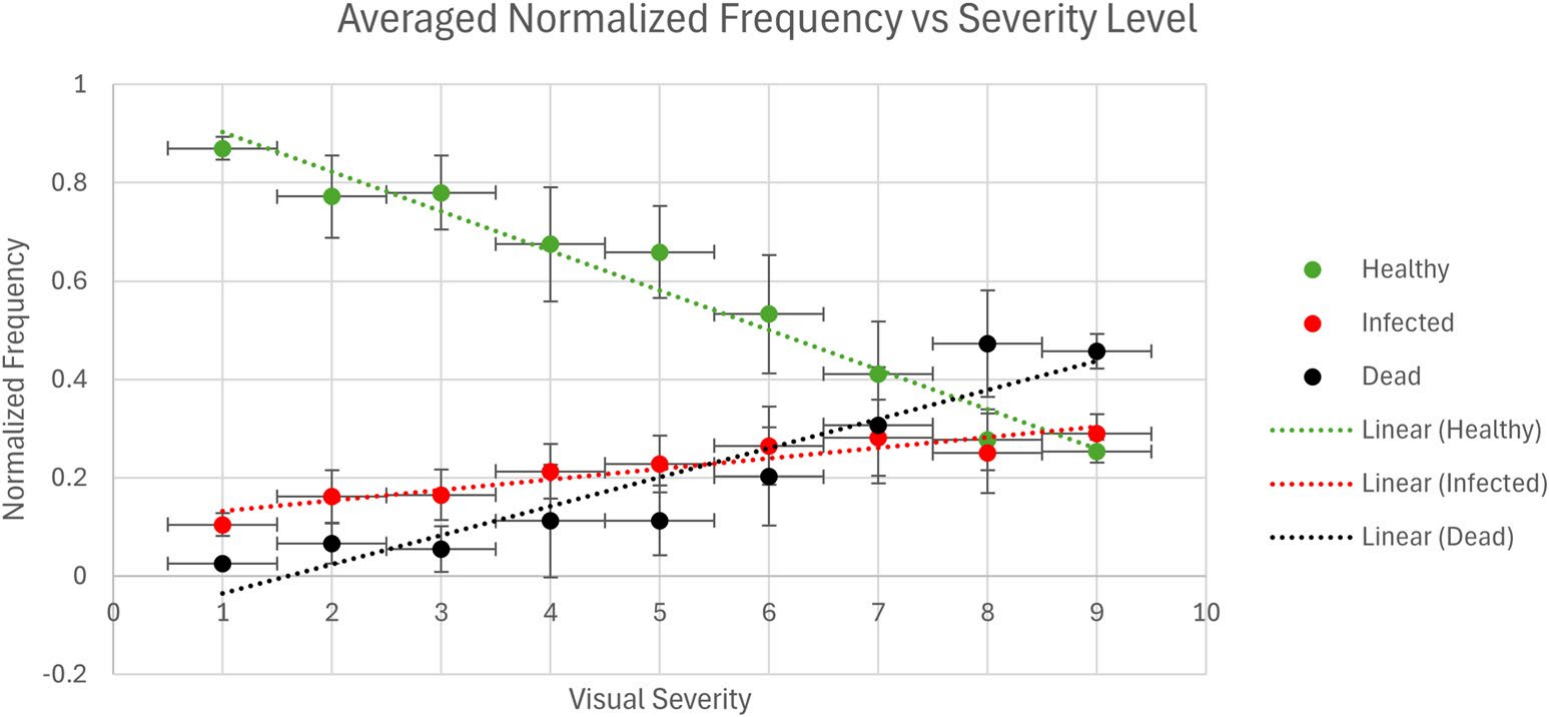
Normalized frequency of Healthy, Infected, and Dead (defoliated) plant material. Vertical error bars represent 1 standard deviation from the mean and horizontal error bars represent half of a visual level

**Fig. 8:**
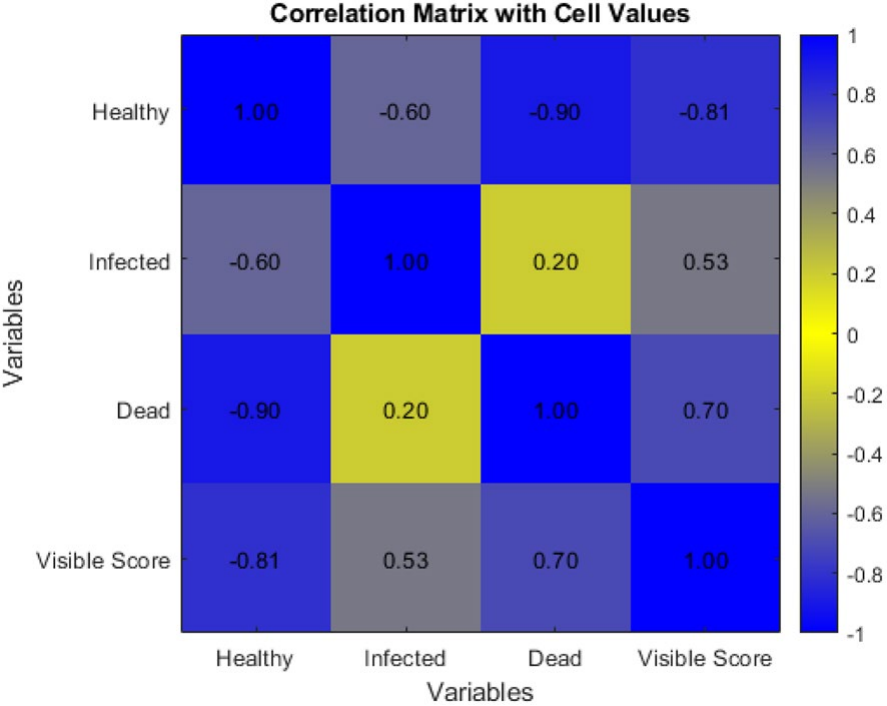
Correlation matrix for three class labels and the visual severity level

To examine the relationship between our two primary classes and visual severity level, a simple data set was created. This data set, referred to as the *Image Scoring Data Set*, was comprised of 475 images captured at the end of the growing season on 5 Oct. 2022 at PBRS per Table 1. Each of the full-size 12MP images in the *Image Scoring Data Set* was segmented by our network and the labels were converted into their relative class frequency. Each visual severity level represents a small range of possible severities—for example, a score of 4 is described as having “many spots; mostly on lower and middle leaves; disease evident” [11]. This description represents a range of severities before increasing to the next score, effectively introducing quantization or round-off error to the measurements [48]. To reduce error caused by this range, all samples of a specific visual severity were averaged to a single value, representing the typical infection for each visual severity score. Figure 7 shows a plot of the average normalized frequency of each of these class counts as compared to the visual severity level. The $r^2$ for this fitting can be found in Table 7 alongside the root-mean-square-error (RMSE) when the fit function is applied to all images in the *Image Scoring Data Set*. A fit was performed on the classification frequency to the linear function $f = p_{00} + p_{01} s_v$ where *f* is the normalized frequency, $s_v$ is the ground truth visual severity level, $p_{00}$ is an initial offset, $p_{01}$ is the multiplication coefficient for the visual severity level. The results of fitting to a linear trend as described here can be seen in Table 7. A continuous decrease in the Healthy plant frequency is observed as the visual severity level increases. We also see an increase in Infected plant material and Dead plant material as the severity increases. A correlation coefficient matrix can be seen in Figure 8, showing the correlation coefficients of each pixel class and the visual level.

**Table 7:** Parameters of fits for normalized class frequency alongside quality of fit metrics

	$p_{00}$	$p_{01}$	RMSE	Average severity $r^2$
Healthy green area	0.984	− 0.081	0.109	0.960
Infected leaf area	0.110	0.022	0.0687	0.883
Dead plant material	− 0.094	0.059	0.1068	0.883

Using the *Image Scoring Data Set*, the ratios of Infected and Dead pixels within the image can be used as inputs and converted into a predicted visual score by

1 $$\begin{aligned} s_p = 1.0 + 11.2621\delta + 7.6794\omega , \end{aligned}$$

where $\delta$ is the normalized frequency of Infected pixels, $\omega$ is the normalized frequency of dead pixels, and $s_p$ is the predicted visual level. This equation was determined by performing a linear regression across all samples in the *Image Scoring Data Set*. The Healthy classification ratio was not needed as it is linearly dependent upon the Infected and Dead values.

Using this linear regression equation, we can determine a new level that is directly comparable to the 9-point scale. By converting from the normalized frequency of plant classes to the 9-point scale, it can be determined how well this image scoring system replicates the results of our visual severity levels. Using the *Image Scoring Data Set*, the visual severity level can be estimated from the ratio of Infected and Dead pixels 1. As mentioned previously, occasionally more images were taken per plot, which can lead to an imbalance in the scored data set as not all plots were imaged equally. To eliminate this possible bias, a random subsampling of images was performed. Only three images per plot were selected, and each image was passed through the scoring pipeline to determine an estimated leaf spot severity rating. The root mean square error (RMSE) value was calculated to be 0.996. Some of this error can be attributed to the 9-point scale’s integer basis compared to the continuous levels of the predicted level. While the visual severity levels force a small range of infection severities to be equal to a single integer level, the predicted level is continuous and will not always match up exactly with the original level.

To identify how the number of images and sampling area impacts the expected error, a second comparison was performed where the predicted level of three images per plot, the same randomly sampled images described previously, were averaged before being passed into the scoring function. The resulting predicted score was then compared to the ground truth measurement for the plot as a whole. This increases the area of the plot that is being measured by a factor of three. When performing this average across three images the RMSE for each plot decrease to approximately 0.800 from the previous 0.996.

Additionally, non-linear fitting functions were investigated to determine the suitability of the linear function. Three support vector machines (SVM) were fit to the entire *Image Scoring Data Set* using the Regression Learner from MatLab 2023b. The kernel scale, box constraint, and epsilon were all determined dynamically by the Regression Learner application. The Linear SVM was found to be the highest performing with an RMSE of 1.064, followed by the Cubic SVM with an RMSE of 1.071, and lastly was the Quadratic SVM with an RMSE of 1.081. Due to the performance of the SVM models all being slightly worse than the linear model, the linear model was selected as the final model for converting segmentation values into an estimated leaf spot score.

### Predicted severity over time

**Fig. 9:**
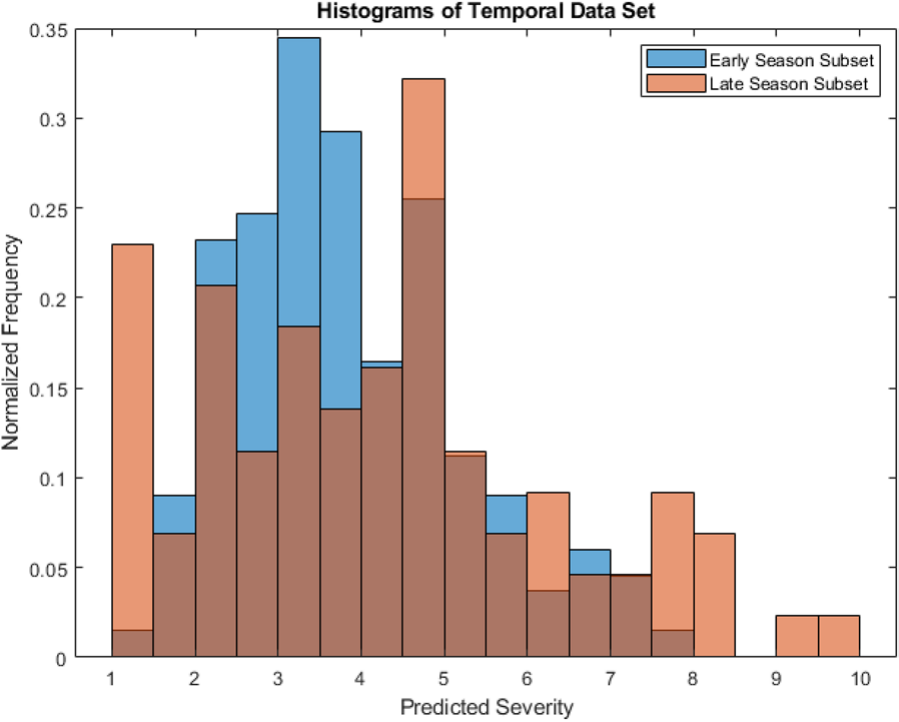
Histograms of randomly sampled plots at two different points in time

One limitation of our study is that visual severity levels were only conducted at the end of each growing season; however, image data were collected at earlier time-points with our scoring protocol, our previously described *Temporal Data Set*. A simple hypothesis to test is whether the scoring algorithm produces a lower average level early on in the season, when leaf spot severity should be lower due to the reduced duration of the infection period [5]. To quantify trends in the data over time, our temporal dataset was used to predict the distribution of levels from the UCPS location at two different points in time toward the end of the 2022 growing season (exact dates can be found in Table 1). To avoid biasing the data, a random subsampling was performed in the cases where more than three images were captured of a particular plot, resulting in each of our plots in the data set contributing three images to the analysis. A graph of normalized frequency versus predicted levels for both sampling dates can be seen in Fig. 9. The mean predicted level for the early season subset was 3.87 where the mean predicted level for the late season subset was 4.29, an increase of 0.42 over the course of 12 days. A wider spread of levels is observed on the late season subset, with the standard deviation of 1.33 on the early subset and a standard deviation of 2.18 on the late subset. Finally, we see a higher maximum predicted level in the late subset; the maximum predicted level being 9.32 in the late subset and 7.71 in the early subset. The minimum predicted level in the early subset is 1.05 compared to 1.11 in the late subset.

## Discussion

### Handheld imaging protocol and dataset creation

The first step in this automated leaf spot scoring pipeline involved the handheld imaging component. As described previously, our imaging personnel used a cell phone camera, combined with a simple handheld tool, to perform rapid imaging of a random selection of plots. For our purpose, we needed to obtain hundreds of images across numerous plots in our field trials. Compared to a traditional visual scoring, our imaging protocol was more time-intensive than traditional expert visual scoring by a factor of five. While this limitation may mean that field surveys require five times as many personnel to survey at the same rate as an individual using traditional field survey protocols, the training time is reduced with the imaging protocol and scalability is increased. The element of human subjectivity present in traditional field surveys is greatly reduced using our protocol, which reduces the error introduced by having more personnel present in the scoring process. An additional benefit is that the raw data is stored in the form of captured images, where a traditional field survey does not store data that can easily be revisited if errors are identified.

When the plots were imaged, a minimum of three images were taken, two at either end and one in the center. This does not fully image the plot and means that the entire 6.8 square meter plot must be scored on a combined area of 2.7 square meters, a reduction in area scored by 60%. As described in the results, an RMSE of 0.996 visual scores was observed when evaluating the individual images in the *Image Scoring Data Set*, and when randomly averaging the results of three images, the RMSE was reduced to 0.800. This reduction in error indicates that a higher number of images reduces the error seen when evaluating a plot for infection severity. This is reinforced by the $r^2$ values when fitting the normalized frequency of each class per the severity score, as seen in Fig. 7 and Table 7. Here, we see a high $r^2 \approx 0.88-0.95$ for all classes when averaging all images associated with a particular severity score. As previously described, the discrete nature of the 9-point scale and the continuous output of the scoring function introduces round-off errors into the measurements. Representing a range of infection severities as an integer value causes unavoidable error due to compressing each severity range to a single integer. Round-off error in the ground truth measurements leads to an unavoidable error when evaluating the performance of the pipeline.

As a limitation of this study, only three images were captured per plot, which limits the multi-angle coverage of each area and could result in leaf occlusion. This imaging protocol was performed to minimize time spent capturing images and minimize the barrier of entry. More advanced image collection protocols are under investigation by peanut breeding teams around the country, such as automated drone image capturing pipelines; future work should could investigate the possibility of combining the pipeline developed here with these systems that are capable of more fully imaging plots and minimizing leaf occlusion [20]. Future work could examine integrating the pipeline developed here with such systems to achieve more comprehensive plot imaging and reduce the impact of leaf occlusion.

### Neural network and scoring algorithm

A key neural network metric is how predictably it performs on images associated with specific levels of infection severity. The network must both classify pixels correctly and fit into the overall scoring pipeline. One key identifiers of the semantic segmentation network’s generalizability is the normalized confusion matrix that was generated from the testing (holdout) subset of the *Neural Network Development Data Set* as seen in Fig. 6. These results demonstrate the varying degrees of accuracy for the four classes. The least important class—Non-Plant—shows the highest degree of accuracy. The Healthy and Infected classes show similar levels of accuracy, with accurate classification $>80.0$% of the time. The Dead class was frequently mistaken for the Non-Plant class, with 40.0% of the Dead pixels being mistaken for Non-Plant pixels and only 53.8% of the Dead class being identified correctly. Despite having the second-highest mean accuracy across all classes, the ResNet50-based model had the worst identification of Non-Plant material of any of the four models. The next closest performer in mean accuracy was MobileNetV2. However, this model suffered from a low accuracy (66.5%) in identifying infected material. ResNet18 performed poorly across almost all classes, with Dead pixel classification accuracy of only 18.5% and Infected pixel classification accuracy of 70.4%. The U-Net model performed similarly to ResNet50 on both the Healthy and Infected classes, and even outscored ResNet50 in regards to the Non-Plant class, but suffered from a low accuracy of 34.9% on the Dead class, indicating that it may struggle in identifying one of the key classes to our scoring pipeline. Finally, the Jaccard index, shown in Table 6, was another key metric for success when evaluating the networks tested for developing our pipeline. The ResNet50 model scored the highest Jaccard Index of the four networks created. While the mean accuracy was slightly lower than the MobileNet-based model, the higher Jaccard Index indicated that the labels produced by the ResNet50 model were more similar to the semantic labels created for testing compared to the labels produced by any other model. The Jaccard Index is based on the overlapping area of the labels rather than a raw accuracy of correctly applied labels; this behavior means the metric is less impacted by imbalanced datasets than the overall accuracy metric, making it key to evaluating the performance of the trained neural network [49].

Because many other damage detection pipelines treat damage as a singular class rather than differentiating between different classifications of damage, the networks’ ability to differentiate between damage to the canopy in the form of infected lesions and dead plant material was of key interest [50]. In the ResNet50 model that was chosen, Infected pixels were misclassified as Dead 5.7% of the time, and Dead pixels were misclassified as Infected 1.6% of the time. Other models also showed a low misclassification rate between the two different damage classifications. The ResNet18 model for example showed no misclassification where Infected was mislabelled as Dead, and only 3.6% of Dead pixels were misclassified as Infected; the U-Net and MobileNetV2 also showed low misclassification rates between Infected and Dead, as seen in Fig. 6. These results are indicative that the four models tested are capable of differentiating between the types of damage seen in our field trials, implying suitability for these networks in multi-class classification tasks across different timespans. Future work may focus on developing pipelines capable of detecting and scoring additional types of damage in peanut, to allow for a more complete tool.

We saw a small level of defoliation that was present even at low levels of severity infection. Referring to Fig. 6, we observed that Non-Plant was mistakenly for Dead plant material 2.5% of the time. The average frequency of Dead plant material in an image associated with a level 1 severity infection was 0.03, or approximately 3% of pixels classified as plant material. The standard deviation of our Dead pixel frequency from images associated with a level 1 infection was only 0.01, or 1%. It is believed that much of this remaining error in Dead classified pixels can be attributed to misclassification by the neural network; the confusion matrix seen in Fig. 6 shows that 40% of the Dead pixels in the holdout set were misclassified as Non-Plant. Much of the Non-Plant was soil, which indicated that many of our Dead pixels were mistaken for soil or other background material. Despite the misclassification, the dead class showed a strong linear relationship with the visual ground truth score, as shown in Fig. 7. The averaged dead classified pixel fit demonstrated an $r^2$ of 0.883 and an RMSE of 0.11 as detailed in Table 7. This indicated that, while misclassification on the full Score Set may be higher than that of the test subset from the *Neural Network Development Data Set*, it was low enough and consistent enough for use in the scoring pipeline. This predictable behavior was reinforced by explicitly quantifying the Healthy class’s linearity, where the average fitting function had an $r^2$ of 0.960 when predicting the visual score as seen in Table 7. The RMSE values were on the order of magnitude of 0.1, indicating a variance of almost 10% of the pixels within an image versus the linear function’s prediction. Given that each severity score represents a range of infection severity, this range of possible outputs within a predicted score is to be expected. A high correlation coefficient between the visual score and the healthy number of pixels, with a correlation coefficient of − 0.81. It should be noted that the three normalized classification values are linearly dependent on one another; the healthy pixel value is equivalent to $1-\delta -\omega$, so while the Dead and Infected classes suffered from a lower correlation, the combined sum of the two provided a strong correlation to the visual severity score that was equivalent to the healthy class score.

The overall pipeline had an RMSE of 0.996 visual scores when using individual images and 0.800 visual scores when using three images per plot. The average error for an image will be just under one level of severity on the 9-point scale, while imaging more of the plot and averaging the scores of several images can reduce the error to below one severity level, indicating the suitability of the pipeline.

The pipeline includes an intermediary step to extract ratios of Healthy, Infected, and Dead plant regions. While the 9-point scale is used by NC State’s breeding program, other scales, such as that of Chiteka et al. have been historically applied [6, 11, 20]. If the breeding community adopts a new scale, the neural network can still be used, requiring only adjustments to the scoring function to accommodate the new severity standard.

### Predicted score over time

The randomly sampled scores over time served as a demonstration of the pipeline’s scalability. Given this pipeline’s purpose as a tool for monitoring severity in peanuts, it is important to show that the pipeline can be used not just to score at the end of field trials (when scoring would normally be done), but also as a tool to monitor the severity’s progression over time, which impacts yield [5]. Using the *Temporal Data Set*, the early season subset demonstrated a significantly lower maximum predicted score on individual images than the late season subset—a maximum score of 7.71 versus 9.32, respectively. A higher mean single image score was also seen, with the mean increasing from 3.87 to 4.29—an increase of 0.42 in the predicted visual score between the two subsets. The late-season subs*et al*so exhibited a higher standard deviation compared to the early-season data set of 2.18 versus 1.33, respectively, which indicated that the spread of predicted severities increased as the growing season progressed.

While these observations aligned with our expectations, the limited number of sampling dates makes it difficult to draw definitive conclusions about the sensitivity of our pipeline to small changes in the severity of the infection over time. However, such a capability would allow for the detection of increasing severities over time and would enable recurring monitoring of leaf spot progression during field trials—offering a new dimension of analysis for peanut breeding programs. While future studies—which capture visual score data at different times of the year—will help to further justify this in the future, these results indicated that capturing a wider range of scores across a larger span of time should be possible using this pipeline.

## Conclusion

This study successfully achieved the main objective of developing an automated peanut leaf spot severity scoring pipeline to augment the subjective visual severity scale commonly used in peanut breeding programs. By employing a ResNet50 architecture, a machine learning model was developed that provides a reliable method to measure the infected and defoliating regions of peanut, allowing for an objective determination of infection severity. The significance of our peanut scoring pipeline lies in its potential to overcome the limitations of human subjectivity and variability associated with manual scoring methods. By automating the scoring process, we ensure consistent and reliable results across different field trials, facilitating collaboration and enabling researchers to compare findings accurately. This pipeline serves as a valuable tool for peanut breeders and scientists, offering a standardized approach to scoring and monitoring leaf spot severity in peanuts.

Moreover, our automated system opens avenues for future collaboration and research. By eliminating the influence of human subjectivity, researchers can confidently compare results, exchange data, and validate findings across different breeding programs. This promotes transparency and efficiency in peanut research, ultimately contributing to the development of improved peanut varieties and disease management strategies.

In conclusion, our peanut scoring pipeline provides a reliable and objective approach to assess leaf spot severity in peanuts. By integrating automated image analysis and machine learning techniques, we offer a valuable tool for peanut breeders and researchers, improving the efficiency, consistency, and accuracy of scoring processes. The pipeline has the potential to revolutionize peanut breeding programs and contribute to advancements in disease management strategies, ultimately benefiting the agricultural community and ensuring the sustainable production of disease resistant peanut.

## Data Availability

The datasets generated and/or analysed during the current study are available in the Automated Leaf Spot Scoring GitHub repository: https://github.ncsu.edu/jclarse2/AutomatedLeafSpotScoring.

## Footnotes

1 GitHub Repository containing all training data.
